# MiR-216a-5p-containing exosomes suppress rTp17-induced inflammatory response by targeting TLR4

**DOI:** 10.1042/BSR20190686

**Published:** 2019-08-07

**Authors:** Rui-Rui Peng, Shu-Xian Shang, Li-Shi Zhao, Fu-Quan Long

**Affiliations:** 1 STD Institute, Shanghai Dermatology Hospital, Tongji University School of Medicine, Shanghai 200443, China; 2 Institute of Dermatology, Chinese Academy of Medical Sciences and Peking Union Medical College, Nanjing 210042, China

**Keywords:** exosomes, MiR-216a-5p, rTp17, TLR4

## Abstract

Syphilis caused by *Treponema pallidum* (*T. pallidum*) infection is accompanied by inflammatory injury of tissue, and has a worldwide distribution and increasing incidence over the past decade. Tp17 has been reported to be a strong membrane immunogen, and was initially observed to play a role in inflammation during syphilis, reacting intensely with human syphilitic sera. We therefore used recombinant Tp17 (rTp17) as a stimulator in our study. Increasing evidence has demonstrated that microRNA (miRNA)-containing exosomes have emerged as a potential effective therapeutic target for many diseases. However, the biological functions and molecular mechanisms of miR-216a-5p in syphilis pathogenesis remain unknown. Our study first identified dramatically decreased miR-216a-5p in plasma of syphilis patients compared with the healthy control, which was negatively correlated with the expression of inflammatory cytokines, including IL-1β, IL-6, and TNF-α. Moreover, endothelial cells treated with miR-216a-5p-containing exosomes significantly attenuated the rTp17-induced inflammatory response. More importantly, we identified that miR-216a-5p could bind to the 3′-untranslated region (UTR) of Toll-like receptor (TLR) 4 (TLR4), and overexpression of TLR4 largely rescued the miR-216a-5p-mediated suppression of rTp17-induced inflammatory cytokine production and the TLR4-MYD88 signaling pathway. Thus, our results reveal a novel role of miR-216a-5p-containing exosomes in endothelial cells, implying a potential therapeutic target for inflammation in syphilis patients.

## Introduction

Syphilis is a multistage, rapidly disseminated disease caused by the spirochetal bacterium *Treponema pallidum* subsp. *pallidum*. Syphilis can result in multisystem involvement with significant morbidity. The disease has a worldwide distribution and has had increasing incidence over the past decade [1–3]. Following inoculation, spirochetes replicate locally and induce an inflammatory response that leads to the distinctive, painless chance of primary syphilis. It has been reported that the Toll-like receptor (TLR) and downstream MYD88 signaling pathway-mediated inflammatory response contributes to syphilis [4]. *T. pallidum* can also attach to and activate vascular endothelial cells and penetrate the intercellular junctions of endothelial cell monolayers [5–6]. However, little is known about the pathogenesis of syphilis because of the inability of *T. pallidum* to be cultured. Studies on the membrane immunogens of *T. pallidum* have provided insight into the immunopathogenesis of syphilis. Among the periplasmic lipoproteins, Tp17 has been reported to be a strong membrane immunogen, and was initially recognized to play a role in inflammation during syphilis, reacting intensely with human syphilitic sera. We therefore used recombinant Tp17 (rTp17) for the stimulator in our study.

Exosomes are a type of secreted vesicle that range in size from 30 to 100 nm in diameter, and are considered mediators of intercellular communication through their cargo. They are also involved in various physiological and pathological processes. Exosomes contain a series of biologically active molecules, such as proteins, DNAs, mRNAs, and microRNAs (miRNAs) [7–9]. miRNAs are short and single-stranded non-coding RNAs that post-transcriptionally regulate gene expression. Currently, increasing evidence has demonstrated that miRNAs are an important therapeutic target for many diseases, including metabolism, inflammation, and cancer [10–13]. MiR-216a-5p has been reported to regulate various cancers through mediating different target genes, such as *Bcl-2, HK2*, and *KLF12* [14–16]. It has also been demonstrated that miR-216a-5p could repress NF-κB pathway activation in H_2_O_2_-induced 16HBE cells via targeting HMGB1 [17]. However, the role of miR-216a-5p in syphilis remains unknown.

In the present study, we first identified that miR-216a-5p decreased in the plasma of patients with syphilis compared with the healthy control, which was negatively correlated with levels of inflammatory cytokines such as IL-1β, IL-6, and TNF-α. We demonstrated the crucial effects of exosomes containing miR-216a-5p on inflammatory cytokine production by endothelial cells. This was done by targeting the TLR4-MYD88 signaling pathway, providing novel insight into miR-216a-5p as a therapeutic target for syphilis.

## Materials and methods

### Patients and clinical characteristics

For the present study, 20 syphilis patients and 20 healthy individuals were selected from Shanghai Dermatology Hospital. Plasma samples from each participant were centrifuged from whole blood (4–5 ml) at 5000×***g*** for 10 min at 4°C and stored at -80°C until further analysis. The study was approved by the Ethical Committee of Shanghai Dermatology Hospital, and informed consent was received from all patients.

### Cell culture and reagents

Human umbilical vein endothelial cells (HUVECs) were purchased from a company named BeNa Culture Collection and cultured in Minimum Essential Medium (Invitrogen), containing 10% fetal bovine serum (FBS), 100 U/ml penicillin, and 0.1 mg/ml streptomycin. Adipose tissue-derived mesenchymal stem cells (ADSCs) were purchased from Cyagen and cultured in Dulbecco’s modified Eagle’s medium (DMEM), supplemented with 10% FBS, 0.1 μM dexamethasone, 50 μM ascorbate-2-phosphate, and 10 mM β-glycerophosphate. The rTp17 protein used in our study was obtained by the methods presented in a previous publication [18].

### Plasmids

The human TLR4 coding region was cloned into the pCDNA3.0 vector. The 3′-untranslated region (UTR) of TLR4 mRNA containing the putative binding site of miR-216a-5p was amplified and cloned into the luciferase reporter psiCHECK2 vector (Promega) to construct wild-type plasmid TLR-3′-UTR^WT^. The mutant 3′-UTR of TLR4 (TLR-3′-UTR^Mut^) with the seed region for the luciferase reporter was obtained using a KOD Site-Mutagenesis Kit (Toyobo, Japan). The sequences of primers for PCR were shown in Table 1 and miRNA mimics or inhibitors were shown in Table 2.

**Table 1:** The sequences of primers for PCR or RT-PCR in the study

Gene/miRNA	Forward primer (5′–3′)	Reverse primer (5′–3′)
psiCHECK2-TLR4-WT	CGAGCTCGCTGAATTCTAAATTGAGATTAGGATCTAAGGACAAGCTTG	CAAGCTTGTCCTTAGATCCTTGAGATTTTTTAGAATTCAGCGAGCTCG
psiCHECK2- TLR4-Mut	CGAGCTCGCTGAATTCTAAATACCACTAGGATCTAAGGACAAGCTTG	CAAGCTTGTCCTTAGATCCTAGTGTGTTTTAGAATTCAGCGAGCTCG
U6	CTCGCTTCGGCAGCACA	AACGCTTCACGAATTTGCGT
miR-216a-5p	AGGCTGGCCGTGATGAATT	GAGAGCCGTGTATGACTCGCT

**Table 2:** The sequences of miRNAs used in the study

miRNA	Sequence (5′–3′)
miR-216a-5p mimics	UAAUCUCAGCUGGCAACUGUGA
miR-216a-5p inhibitor	UCACAGUUGCCAGCUGAGAUUA
Negative control	UUCUCCGAACGUGUCACGUTT

### Exosome extraction

ADSCs were transfected with a plasmid encoding miR-216a-5p using Lipofectamine 2000 (Invitrogen). After 48 h of miRNA transfection, exosomes were isolated from ADSC supernatant using an ExoQuick-TC Kit (SBI) according to the manufacturer’s instructions. The protein content of the exosomes was measured using a BCA™ Protein Assay Kit (Beyotime, Nantong, China).

### ELISA

The secretion levels of the inflammatory cytokines IL-6, IL-1β, and TNF-α were measured in cell supernatant or serum using an enzyme-linked immunosorbent assay kit (Invitrogen) according to the manufacturer’s instructions. Both standards and samples were run in triplicate. The optical density (OD)_450_ was calculated by subtracting the background, and standard curves were plotted.

### Dual-luciferase reporter assay

HUVECs were seeded into 24-well plates and co-transfected with the luciferase reporter TLR-3′-UTR^WT^ or TLR-3′-UTR^Mut^ and negative control or miR-216a-5p mimics (100 nM) using Lipofectamine 2000 (Invitrogen). The cells were harvested 48 h after transfection and the luciferase activities of firefly and *Renilla* were detected using the Dual-Luciferase Reporter Assay System (Promega). Results are presented as the ratio of *Renilla* luciferase activity to firefly luciferase activity.

### Western blotting

HUVECs were lysed in ice-cold RIPA lysis buffer (Beyotime Institute of Biotechnology) to obtain cell lysates. Protein concentrations were measured according to the manufacturer’s manual with the BCA protein assay (Beyotime Institute of Biotechnology). Equal amounts of protein samples were separated on an SDS/PAGE gel and probed with primary antibodies for TLR4 (#14358), MYD88 (#4283), or GAPDH (#2118) overnight at 4°C. An additional incubation was performed for 1 h with an anti-immunoglobin horseradish peroxidase-linked antibody, and the results were visualized with enhanced chemiluminescent (ECL) HRP substrates (Millipore). The band intensity was quantified with Image-Pro Plus 5.1 Image Analysis Software.

### Real-time reverse transcription-PCR

Total cellular RNAs from human plasma and HUVECs were isolated using TRIzol reagent according to the manufacturer’s recommendation (Ambion). One microgram of total RNA was then reverse-transcribed into cDNA with a Reverse Transcription Kit (Takara), followed by cDNA amplification using SYBR Green Master Mix (Takara). The relative expression of target genes was evaluated using a 2^−ΔΔ*C*^_t_ method and normalized to U6.

### Statistical analysis

Data are presented as mean ± SD for three independent experiments. Mean values between two groups were compared using Student’s *t* test. The comparison of means among multiple groups was accomplished by one-way analysis of variance (ANOVA). Correlation analysis was conducted using the Pearson correlation coefficient. *P*-values <0.05 were considered statistically significant. All statistical analyses were performed with GraphPad Prism 8.0.

## Results

### miR-216a-5p level is decreased in syphilis patients and negatively correlated with inflammation

**Figure 1:**
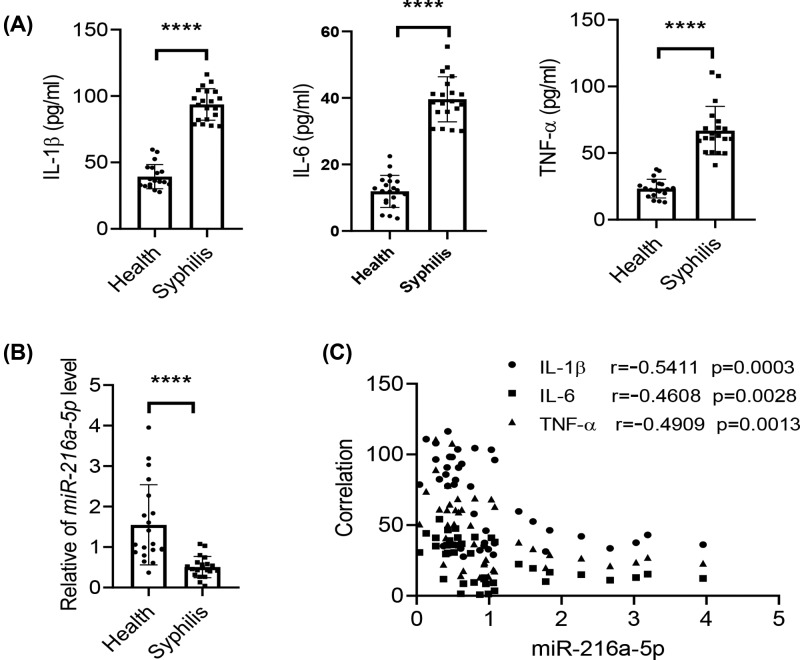
miR-216a-5p level is decreased in syphilis patients and negatively correlated with inflammation (**A**) ELISA analysis of IL-1β, IL-6, and TNF-α in healthy or syphilis-infected serum. (**B**) qPCR analysis of serum miR-216a-5p level. (**C**) The correlation between miR-216a-5p and inflammatory cytokines in serum was evaluated. The results are presented as the mean ± SD. ****P<0.0001.

To evaluate the potential role of miR-216a-5p in syphilis, we collected serum from 20 syphilis patients with *T. pallidum* infection and 20 healthy controls. In accordance with previous studies, the inflammatory cytokines IL-1β, IL-6, and TNF-α were found to be significantly up-regulated in plasma of syphilis patients compared with the healthy controls (Figure 1A). Interestingly, we found that miR-216a-5p expression was strongly decreased in plasma of syphilis patients (Figure 1B). Furthermore, we further evaluated the correlation between miR-216a-5p expression and inflammation. The results showed that the miR-216a-5p expression level was negatively correlated with the expression of inflammatory cytokines IL-1β, IL-6, and TNF-α (Figure 1C), indicating the potential function of miR-216a-5p in syphilis progression.

### Effect of rTp17 on inflammatory cytokine production of HUVECs

**Figure 2:**
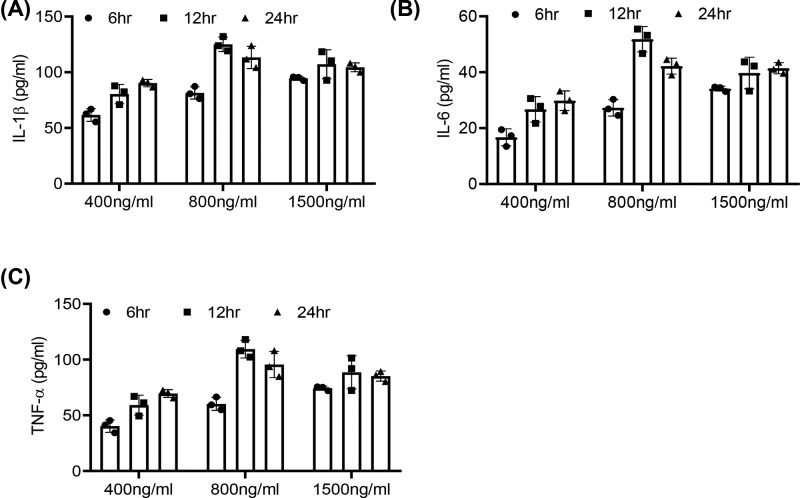
Effect of rTp17 on inflammatory cytokine production of HUVECs (**A**–**C**) Endothelial cells (HUVECs) were treated with rTp17 at different serial concentrations, and inflammatory cytokines such as IL-1β, IL-6, and TNF-α in the cell supernatant were determined via ELISA.

As *T. pallidum* could not be stably cultured *in vitro*, we used rTp17 to stimulate and activate endothelial cells in our study. First, we used different serial concentrations of rTp17 to stimulate endothelial cell line HUVECs to optimize the inflammatory response. HUVECs were incubated with concentrations of rTp17 ranging from 400 to 1500 ng/ml over the time course. ELISA was performed to determine the protein secretion level of inflammatory cytokines IL-1β, IL-6, and TNF-α. The results showed a remarkable increase in these cytokines in the group treated with 800 ng/ml rTp17 for 12 h (Figure 2A–C). Therefore, we chose this stimulation condition for the following study.

### miR-216a-5p-containing exosomes attenuate rTp17-induced inflammatory response

**Figure 3:**
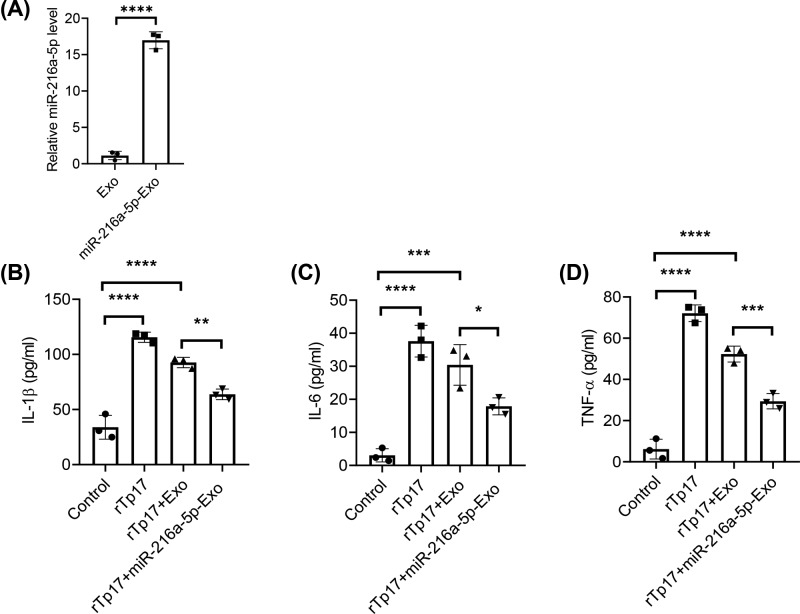
miR-216a-5p-containing exosomes attenuate rTp17-induced inflammatory response (**A**) qPCR analysis of the miR-216a-5p expression level in exosomes. (**B**–**D**) HUVECs were treated with negative control Exo or miR-216a-5p exosome, accompanied with 800 ng/ml rTp17 for 12 h. Inflammatory cytokines such as IL-1β, IL-6, and TNF-α were then evaluated in the cell supernatant via ELISA. The results are presented as the mean ± SD. *P<0.05, **P<0.01, ***P<0.001, ****P<0.0001.

Various studies have illustrated that miRNA-containing exosomes play an important role in the treatment of many diseases, especially in inflammation inhibition. In order to study the function of miR-216a-5p-containing exosomes on the rTp17-mediated inflammatory response, we overexpressed miR-216a-5p in ADSCs and then extracted exosomes to stimulate cells. We first performed qPCR to verify that the miR-216a-5p expression level was indeed up-regulated and enriched in the miR-216a-5p-containing exosomes compared with control exosomes. The stimulation of HUVECs with 800 ng/ml rTp17 for 12 h consistently and significantly promoted the production of inflammatory cytokines IL-1β, IL-6, and TNF-α. The addition of miR-216a-5p-containing exosomes largely decreased the effects of the rTp17-induced inflammatory response compared with the control group (Figure 3A–D). These results demonstrated the negative impact of miR-216a-5p on rTp17-induced inflammation.

### TLR4 is the downstream target of miR-216a-5p

**Figure 4:**
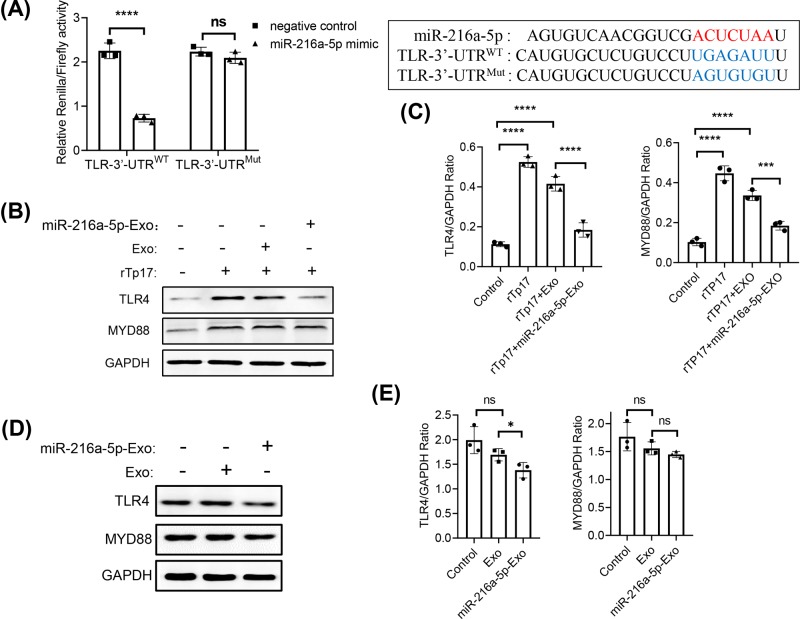
TLR4 is the downstream target of miR-216a-5p (**A**) Predictive binding sites of miR-216a-5p on TLR4 gene (upper). Relative luciferase activity was analyzed in HUVECs co-transfected with TLR-3′-UTR^WT^ or TLR-3′-UTR^Mut^ and miR-216a-5p mimics or negative control (down) (**B,D**). TLR4 and downstream MYD88 expression were determined by Western blot analysis in HUVECs with negative control Exo or miR-216a-5p-containing exosome, with or without 800 ng/ml rTp17 treatment. Quantitative analysis was shown in (**C,E**). The results are presented as the mean ± SD. *P<0.05, ***P<0.001, ****P<0.0001.

To determine how miR-216a-5p negatively regulates the rTp17-mediated inflammatory response, we used bioinformatics analysis (http://www.genecards.org) to predict the possible downstream target of miR-216a-5p. We found a potential binding site of miR-216a-5p on the 3′-UTR of the *TLR4* gene. We thus constructed the 3′-UTR of TLR4, and inserted a point mutation into the site to prevent binding. This is shown in Figure 4A. The relative luciferase activity of HUVECs transfected with TLR4 containing either wild-type or mutated 3′-UTR (TLR-3′-UTR^WT^ or TLR-3′-UTR^Mut^) showed that TLR4 activity was dramatically suppressed by wild-type miR-216a-5p mimics, but not by its mutant form, which lost the ability to be targeted by miR-216a-5p. Studies have reported that TLR4 is one of the key receptors to sense pathogens via the active downstream inflammatory response through the MYD88 pathway, which is also essential for the clearance of *T. pallidum*. We therefore questioned whether miR-216a-5p could target TLR4 to reduce production and downstream MYD88 signaling upon *T. pallidum* infection. Notably, rTp17 stimulation of HUVECs markedly increased the TLR4 expression level, as well as increasing expression of downstream MYD88. Importantly, the administration of miR-216a-5p-containing exosomes significantly decreased the expression of TLR4 and downstream MYD88 (Figure 4B,C). To determine whether Exo and miR-216a-5p-Exo alone have an impact on TLR4/MYD88 expression without Rtp17 stimulation, we treated HUVECs with only Exo or miR-216a-5p-Exo. There was no significant difference in MYD88 expression compared with the control group. Importantly, we observed that miR-216a-5p-Exo also displayed an inhibitory effect on TLR4 expression but not on the MYD88 protein level. However, this difference was smaller compared with stimulation with Rtp17 (Figure 4D,E). These data suggested that miR-216a-5p could target TLR4 to down-regulate the TLR4-MYD88 signaling pathway.

### Effect of miR-216a-5p mimics and inhibitor on TLR4/MYD88 and inflammatory cytokine expression

**Figure 5:**
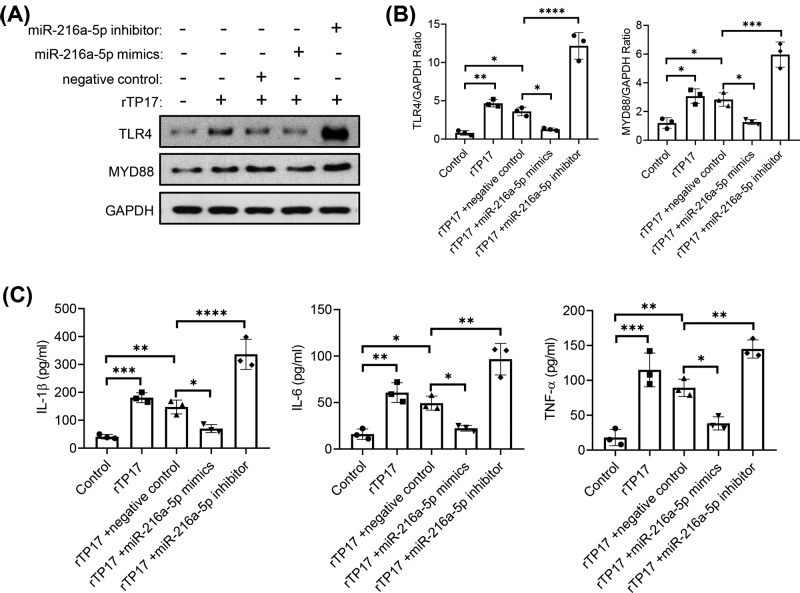
Effect of miR-216a-5p mimics and inhibitor on TLR4/MYD88 and inflammatory cytokine expression HUVECs were treated with negative control or miR-216a-5p mimics and inhibitor accompanied with 800 ng/ml rTp17 treatment. TLR4 and MYD88 expression were analyzed via Western blot assay (**A**). Quantitative analysis was shown in (**B**). Inflammatory cytokines such as IL-1β, IL-6, and TNF-α in the cell supernatant were evaluated via ELISA (**C**). The results are presented as the mean ± SD. *P<0.05, **P<0.001, ***P<0.0001, ****P<0.0001.

Although we confirmed that the miR-216a-5p expression level was up-regulated and enriched in the miR-216a-5p-containing exosomes compared with control exosomes, we could not exclude whether miR-216a-5p-Exo affects cells in other ways. We therefore designed and used the miR-216a-5p mimics and inhibitor to simplify the system. In accordance with the results of exosome treatment, the miR-216a-5p mimics also significantly down-regulated the TLR4 expression level and the corresponding inflammatory cytokine production in rTP17-treated HUVECs compared with the negative control. In contrast, miR-216a-5p inhibitor notably increased TLR4 and cytokine expression (Figure 5A–C). Taken together, our results demonstrated that both miR-216a-5p and miR-216a-5p-Exo have inhibitory roles on TLR4/MYD88 expression and inflammatory cytokine production.

### TLR4 rescues the effect of miR-216a-5p-containing exosomes on rTp17-induced inflammation

**Figure 6:**
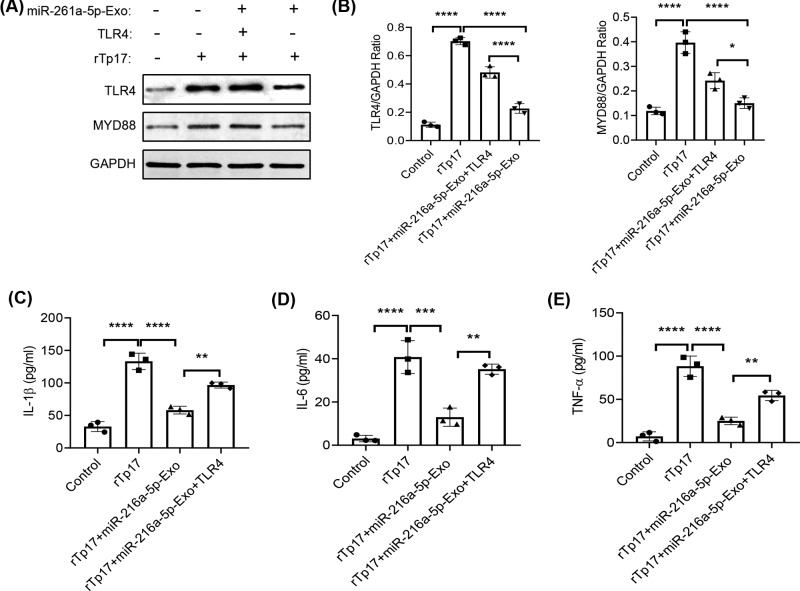
TLR4 rescues the effect of miR-216a-5p-containing exosomes on rTp17-induced inflammation TLR4 was overexpressed in HUVECs and then treated with negative control Exo or miR-216a-5p-containing exosome, accompanied with 800 ng/ml rTp17. We then performed immunoblots (**A**) and quantitative analyses (**B**) of TLR4 and MYD88 expression, as well as analyzing inflammatory cytokines such as IL-1β, IL-6, and TNF-α in the cell supernatant via ELISA (**C**–**E**). The results are presented as the mean ± SD. *P<0.05, **P<0.01, ***P<0.001, ****P<0.0001.

To further confirm that the miR-216a-5p-mediated inhibitory effect on inflammatory cytokine production was dependent on its target TLR4, we constructed a coding region of TLR4 to perform the rescue experiment. First, the immunoblot results showed that miR-216a-5p-mediated down-regulation of TLR4 was completely rescued by TLR4 overexpression, as well as the downstream MYD88 (Figure 6A,B). We then evaluated the effects of TLR4 on the miR-216a-5p-mediated inflammatory response. In accordance with previous studies, miR-216a-5p-containing exosomes dramatically suppressed rTp17-induced inflammatory cytokine production, including IL-1β, IL-6, and TNF-α. Additionally, TLR4 overexpression significantly rescued cells from this suppressive effect (Figure 6C–E). Taken together, our results demonstrated that in endothelial cells, miR-216a-5p-mediated suppression of the rTp17-induced inflammatory response is largely dependent on its target TLR4.

## Discussion

Syphilis is a chronic infectious disease caused by a spirochete, usually venereal in origin, but often congenital. Syphilis can affect almost any body organ if left untreated, and the incidence of syphilis is increasing in most parts of the world. Accumulating evidence has shown that an infection-mediated inflammatory response contributes to the development of syphilis [1–3]. Thus, inhibition of inflammation may be a potential effective therapy strategy for treating syphilis.

Exosomes have been reported to play a role in their therapeutic effect in many diseases [10–13], such as inhibiting inflammation through delivery of therapeutic miRNA. MiR-216a-5p has been acknowledged as an oncogene, and is known to be involved in the progression and metastasis of numerous cancers [14–16]. However, little is known about its role in inflammation or syphilis. To the best of our knowledge, the present study is the first to report that the level of miR-216a-5p in human syphilis was decreased compared with the healthy control. Moreover, there was a negative correlation between the levels of miR-216a-5p and inflammatory cytokines such as IL-1β, IL-6, and TNF-α, which were dramatically increased in syphilis patients. Furthermore, our study revealed that miR-216a-5p-containing exosomes significantly suppressed rTp17-induced inflammatory cytokine production.

It has been reported that miRNAs modulate gene expression by targeting the 3′-UTR to inhibit translation. Notably, our bioinformatics analysis showed that miR-216a-5p can target the 3′-UTR of TLR4, which was confirmed via the TLR4 binding-site mutant in a dual-luciferase assay. TLR4 plays an important role in various infection-induced inflammatory responses, and participates in various cellular functions. Our data revealed that TLR4 overexpression rescues the effect of miR-216a-5p on inflammation, which further confirmed the targeting relationship between miR-216a-5p and TLR4 during the rTp17-mediated inflammatory response.

In summary, our results showed that the level of miR-216a-5p in plasma was significantly reduced in syphilis patients, which was negatively correlated with inflammatory cytokine production. Furthermore, miR-216a-5p-containing exosomes suppressed the rTp17-induced inflammatory response, decreasing IL-1β, IL-6, and TNF-α secretion through targeting TLR4 and the subsequent MYD88 signaling pathway. Our study provides insight into the role of miR-216a-5p in syphilis.

## Abbreviations

ADSC: adipose tissue-derived mesenchymal stem cell
ANOVA: analysis of variance
FBS: fetal bovine serum
GAPDH: Glyceraldehyde-3-Phosphate Dehydrogenase
HMGB1: High-Mobility Group Box 1 Protein
HUVEC: human umbilical vein endothelial cell
miRNA: microRNA
MYD88: Myeloid Differentiation Primary Response Gene 88
qPCR: Real-time Quantitative PCR Detecting System
RIPA: Radio-Immunoprecipitation Assay
rTp17: recombinant Tp17
TLR: Toll-like receptor
*T. pallidum*: *Treponema pallidum*
UTR: untranslated region
